# SPOT REGION OF INTEREST IMAGING: A NOVEL FUNCTIONALITY AIMED AT X-RAY DOSE REDUCTION IN NEUROINTERVENTIONAL PROCEDURES

**DOI:** 10.1093/rpd/ncz290

**Published:** 2020-01-16

**Authors:** Ljubisa Borota, Andreas Patz

**Affiliations:** 1 Department of Surgical Sciences, Uppsala University, Sweden; 2 Canon Medical Systems Europe BV, Zilverstraat 1, 2718 RP, Zoetermeer, The Netherlands

## Abstract

*Aim of the study*: The aim of this study was to describe a new functionality aimed at X-ray dose reduction, referred to as spot region of interest (Spot ROI) and to compare it with existing dose-saving functionalities, spot fluoroscopy (Spot F), and conventional collimation (CC). *Material and methods*: Dose area product, air kerma, and peak skin dose were measured for Spot ROI, Spot F, and CC in three different fields of view (FOVs) 20 × 20 cm, 15 × 15 cm, and 11 × 11 cm using an anthropomorphic head phantom RS-230T. The exposure sequence was 5 min of pulsed fluoroscopy (7.5 pulses per s) followed by 7× digital subtraction angiography (DSA) runs with 30 frames per DSA acquisition (3 fps × 10 s). The collimation in Spot F and CC was adjusted such that the size of the anatomical area exposed was as large as the Spot ROI area in each FOV. *Results*: The results for all FOVs were the following: for the fluoroscopy, all measured parameters for Spot ROI and Spot F were lower than corresponding values for CC. For DSA and DSA plus fluoroscopy, all measured parameters for Spot ROI were lower than corresponding parameters for Spot F and CC. *Conclusion*: Spot ROI is a promising dose-saving technology that can be applied in fluoroscopy and acquisition. The biggest benefit of Spot ROI is its ability to keep the entire FOV information always visible.

## INTRODUCTION

Endovascular treatment of cerebral vascular diseases has developed tremendously during the last 30 years, which has led to a change in treatment strategy and an improvement of the outcomes of treatment of these diseases. In addition, thanks to numerous newly developed endovascular methods of treatment, it is possible to treat some cerebral vascular diseases which were untreatable only 15 or 20 years ago. However, long fluoroscopic times and frequently repeated angiographies, necessary for obtaining high-quality visual information, inevitably carry the risk of the skin damage caused by ionizing radiation^(1–6)^.

Many systems aimed at direct or indirect X-ray protection have been developed. Among them, beam collimators of various forms and constructions were one of the first devices for reducing X-ray dose and have been used almost since the beginning of the X-ray era^(7,8)^.

Though very effective, conventional collimation (CC) has considerable disadvantages:

1. Only symmetric collimation is possible, which leads to an unnecessary exposure for larger anatomical areas than actually needed.
2. Anatomical and/or device-relevant reference information is lost.
3. Patient skin dose is increased when collimating inside the automatic brightness control region of interest (ABC ROI)^(7)^.

CC uses the standard ABC technique with a constant flat panel detector dose, fixed ABC sensing area in size and position, as well as a blade rejection for brightness calculation^(7)^. Besides the CC, several other functionalities aimed at optimizing data acquisition and image post-processing and thus reducing the radiation dose have been developed during recent years.

Söderman *et al.* described a new system for optimization of data acquisition and image creation, which is integrated in the Philips ‘Allura’ biplane angiographic machine^(9)^. Han *et al.* showed that the use of special protection can lead to considerable dose reduction^(10)^.

**Figure 1:**
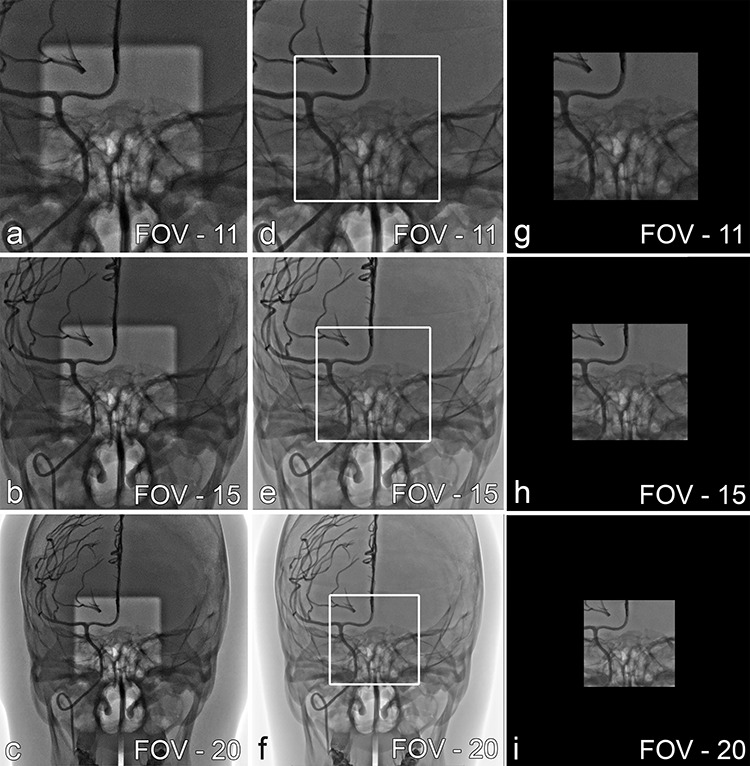
(**a–c**) Spot ROI. The brighter square in the center indicates the ROI exposed with a normal dose. The darker surrounding area indicates the area with additional attenuation of 0.7 mm Cu; (**d–f**) Spot F. The white frame in the center indicates the ROI exposed. Surrounding area is shielded by collimation with LIH superimposed; (**g–i**) CC

According to Khan *et al.*, dose reduction can be achieved despite prolonged intervention time if modification of default settings on biplane angiography equipment is applied^(11)^. Borota and Patz have shown that improved fluoroscopy based on a new construction of ABC as well as flexibility of lateral isocenter of a biplane angiographic machine significantly contributes to the reduction of the dose^(12,13)^.

**Figure 2:**
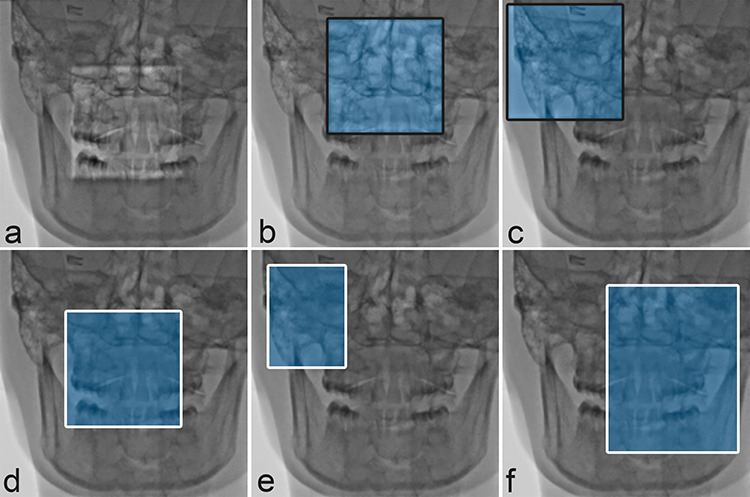
(**a–c**) Spot ROI. Blue transparent area illustrates the ABC sensing area adaptation—not visible in reality. (a) Spot ROI in center position; (b and c) Spot ROI in off-center position; (**d–f**) Spot F; white frame delineates the collimation applied. The LIH is superimposed over the collimator blades. Blue transparent area illustrates the ABC sensing area adaptation—not visible in reality. (d) Spot F with centric and symmetric collimation. (e and f) Spot F with acentric, asymmetric collimation

The development of spot fluoroscopy (Spot F) some years ago has greatly improved the situation as it permits acentric, asymmetric collimation^(12)^. However, as it is still based on collimation, the entire field of view (FOV) anatomy is not visible, and some anatomical and/or device-relevant information is still hidden (Figures 1 and 2). Spot region of interest (Spot ROI), a novel functionality, has been developed and recently become available for reduction of X-ray dose during neurointerventional interventional procedures. The functionality, as well as Spot F, is integrated into the commercially available new ‘Alphenix’ biplane angiographic machine designed and manufactured by Canon Medical Systems (Canon, formerly Toshiba Medical Systems, Tochigi, Japan) (Figures 1 and 2).

The Spot ROI technology is based on two fundamental novel elements:

1. A new type of collimator with an integrated additional 0.7 mm copper filter with a square shaped whole of a defined size in it. The filter can be activated and used in addition to the standardly integrated Cu filter (0.2; 0.3; 0.5 mm) at any time by the user. It can be freely moved in any X–Y direction from table side. The operator can move it either under fluoroscopic control or virtually on the last image hold (LIH). This design permits a ROI exposure with normal dose, while the dose to the surrounding anatomy is significantly reduced thanks to the higher attenuation of the additional copper filter. This enables the user to conveniently position the ROI always over the vascular structure of interest independently of its location within the FOV selected. Therefore Spot ROI provides always full FOV information. As the Spot ROI is of a fixed size, the anatomical area exposed is constant, but its displayed size varies over the different FOVs following the same geometrical magnification rules (Figure 1a–c).
2. Novel ABC technique which in contrast to the conventional ABC technique uses an adaptive ABC ROI instead of a static ABC ROI^(14)^. As the Spot ROI filter can freely be moved, the ABC ROI needs to accurately track the position of the Spot ROI in real time in order to ensure a correct brightness detection and interpretation, which is an essential precondition for the dose-saving effect. Since the backscatter is considerably reduced by the additional 0.7 mm Cu around the Spot ROI, the detector input dose is reduced by a certain percentage to maintain a signal-to-noise ratio similar to that achievable without Spot ROI and higher backscatter.

The aim of this study was to compare the dose impact of the novel Spot ROI functionality with the existing dose-saving techniques CC and Spot F.

## MATERIAL AND METHODS

**Figure 3:**
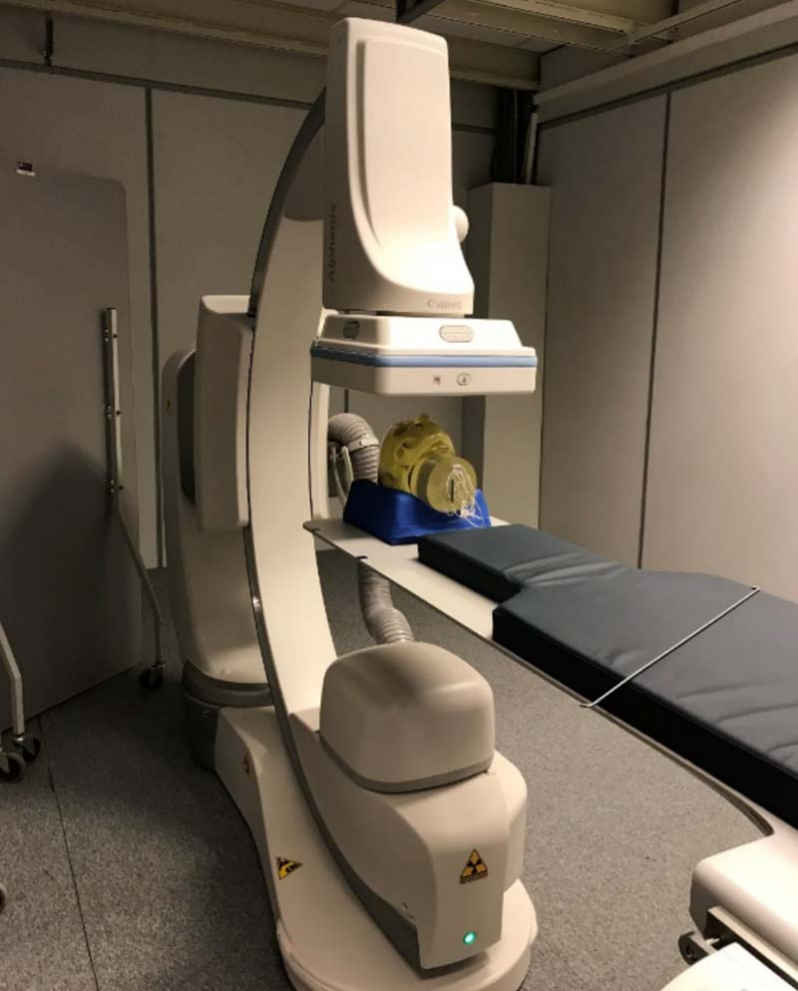
C-arm with an anthropomorphic RS-230T head phantom placed on the table

An anthropomorphic head phantom RS-230T was used as a target (Figure 3). The system was equipped with a 30 × 30 cm a Si flat panel detector (TFP1200C/A1.OEM, Varian Medical Systems, CA, USA) with a pixel size of 194 μm.

Table height was 105 cm; source to image distance was 100 cm with the C-arm in PA orientation. The exposure sequence was 5 min of pulsed fluoroscopy (7.5 pulses/s), followed by 7× digital subtraction angiography (DSA) runs with 30 frames per DSA acquisition (3 fps × 10 s). The collimation in Spot F and CC was adjusted such that the size of the anatomical area exposed was as large as the Spot ROI area in each FOV (Figure 2). Dose area product (DAP) was measured using the built-in DAP meter Diamentor K1/K2 (PTW Freiburg, Germany) for all fields of view.

The air kerma (AK) value is automatically calculated by dividing the DAP by the area exposed at the interventional reference point (IRP) and displayed on the system monitor. It is a standard built-in function of any angiographic system. Peak skin dose (PSD) was used to identify and display the patient skin surface area with the highest cumulative dose delivered. It uses system-specific calibration data and computation algorithm for the estimation of the PSD value. The accuracy tolerance of PSD estimation has been proven to be ~10% which is in fact more accurate than the usual DAP meter accuracy with a tolerance of 35%^(15)^.

The technical characteristics of pulsed fluoroscopy used for the measurements were detector input dose of 0.041 μGy per frame for a reference FOV of 20 × 20 cm and 0.061 μGy per frame for reference FOVs of 15 × 15 cm and 11 × 11 cm. The beam filter was 0.3 mm Cu for all fluoroscopy acquisitions. For the DSA series, the detector input dose was 1.45 μGy per frame for a reference FOV of 20 × 20 cm and 1.75 μGy per frame for reference FOVs of 15 × 15 cm and 11 × 11 cm. The beam filter was 0.2 mm Cu for all DSA acquisitions. The X-ray parameters for all acquisitions can be seen in Table 1. DSA acquisitions of the entire FOV for Spot F and CC were performed for two reasons:

1. To create a sufficient patient skin dose map that also permitted a clear visual difference in the total dose applied between the three modalities.
2. To simulate the clinical reality in which DSA is always used after or between fluoroscopic runs to obtain the entire FOV information. Therefore, no collimation was applied in Spot F and CC, while Spot ROI was applied because it always keeps the entire FOV information visible.

**Table 1:** X-ray tube parameters, beam filters, and results of measurements of AK, DAP, and PSD for fluoroscopy, digital subtraction angiography, and digital subtraction angiography plus fluoroscopy for FOV = 11 cm.

**Fluoroscopy**												
	*FOV*	*Effective field size exposed (cm)*	*SID*	*FS (mm)*	*P/s*	*kV*	*mA*	*ms*	*Beam filter*	*AK*	*DAP*	*PSD*
										*mGy*	*cGycm^2^*	*mGy*
SR	11	8 × 8	100	0.4	7.5	75	106	11.4	0.3Cu	43.5	170.3	59
SF	11	5 × 5	100	0.4	7.5	75	114	11.7	0.3Cu	98.56	174.03	56
CC	11	5 × 5	100	0.4	7.5	80	109	1	0.3Cu		202.6	70
**DSA**												
	*FOV*	*Effective field size exposed (cm)*	*SID*	*Frame rate (fps)*	*No. of runs*	*kV*	*mA*	*ms*	*Beam filter*	*AK*	*DAP*	*PSD*
										*mGy*	*cGycm^2^*	*mGy*
SR	11	8 × 8	100	3	7	87	160	75.7	0.2Cu	96.31	380.24	140
SF	11	8 × 8	100	3	7	80	400	76	0.2Cu	364.17	1445.39	272
CC	11	8 × 8	100	3	7	80	400	76	0.2Cu	364.17	1445.39	272
**Fluoroscopy + DSA**												
	*FOV*	*Total values*	*AK*	*DAP*	*PSD*							
			*mGy*	*cGycm^2^*	*mGy*							
SR	11	Fluoro + DSA	139.81	550.54	199							
SF	11	Fluoro + DSA	462.73	1619.42	328							
CC	11	Fluoro + DSA	479.44	1648.08	342							

**Abbreviations: SID, source image distance; FS, focus size; P/s, pulse per second.**

**Table 2:** X-ray tube parameters, beam filters, and results of measurements of AK, DAP, and PSD for fluoroscopy, digital subtraction angiography, and digital subtraction angiography plus fluoroscopy for FOV = 15 cm.

**Fluoroscopy**												
	*FOV*	*Effective field size exposed (cm)*	*SID*	*FS (mm)*	*P/s*	*kV*	*mA*	*ms*	*Beam filter*	*AK*	*DAP*	*PSD*
										*mGy*	*cGycm^2^*	*mGy*
SR	15	11 × 11	100	0.4	7.5	75	92	10.3	0.3Cu	23.91	205.95	46
SF	15	5 × 5	100	0.4	7.5	75	95	10.6	0.3Cu	71.69	95.66	47
CC	15	5 × 5	100	0.4	7.5	76	114	12	0.3Cu	106.68	129.65	63
**DSA**												
	*FOV*	*Effective field size exposed (cm)*	*SID*	*Frame rate (fps)*	*No. of runs*	*kV*	*mA*	*ms*	*Beam filter*	*AK*	*DAP*	*PSD*
										*mGy*	*cGycm^2^*	*mGy*
SR	15	11 × 11	100	3	7	86	160	67.6	0.2Cu	62.42	483.37	134
SF	15	11 × 11	100	3	7	81	400	53.1	0.2Cu	250.54	1940.17	201
CC	15	11 × 11	100	3	7	81	400	53.1	0.2Cu	250.54	1940.17	201
**Fluoroscopy + DSA**												
	*FOV*	*Total values*	*AK*	*DAP*	*PSD*							
			*mGy*	*cGycm^2^*	*mGy*							
SR	15	Fluoro + DSA	86.33	689.32	180							
SF	15	Fluoro + DSA	322.23	2035.83	248							
CC	15	Fluoro + DSA	357.22	2069.82	264							

## RESULTS

Our results are summarized in Tables 1–3. The size of FOVs, both nominal and effective (cm) are shown on separate columns for each FOV and both modalities (fluoroscopy and DSA).

**Table 3:** X-ray tube parameters, beam filters, and results of measurements of AK, DAP, and PSD for fluoroscopy, digital subtraction angiography, and digital subtraction angiography plus fluoroscopy for FOV = 20 cm.

**Fluoroscopy**												
	*FOV*	*Effective field size exposed (cm)*	*SID*	*FS (mm)*	*P/s*	*kV*	*mA*	*ms*	*Beam filter*	*AK*	*DAP*	*PSD*
										*mGy*	*cGycm^2^*	*mGy*
SR	20	14.5 × 14.5	100	0.4	7.5	75	81	9.5	0.3Cu	15.56	195.22	37
SF	20	5 × 5	100	0.4	7.5	75	85	9.8	0.3Cu	61.91	87.76	35
CC	20	5 × 5	100	0.4	7.5	76	108	11.4	0.3Cu	92.27	147.54	54
**DSA**												
	*FOV*	*Effective field size exposed (cm)*	*SID*	*Frame rate (fps)*	*No. of runs*	*kV*	*mA*	*ms*	*Beam filter*	*AK*	*DAP*	*PSD*
										*mGy*	*cGycm^2^*	*mGy*
SR	20	14.5 × 14.5	100	3	7	84	160	65.1	0.2Cu	47.86	419.65	121
SF	20	14.5 × 14.5	100	3	7	80	400	40.5	0.2Cu	203.86	2543.3	160
CC	20	14.5 × 14.5	100	3	7	80	400	40.5	0.2Cu	203.86	2543.3	160
**Fluoroscopy + DSA**												
	*FOV*	*Total values*	*AK*	*DAP*	*PSD*							
			*mGy*	*cGycm^2^*	*mGy*							
SR	20	Fluoro + DSA	63.42	614.87	158							
SF	20	Fluoro + DSA	265.77	2631.06	195							
CC	20	Fluoro + DSA	296.13	2690.84	214							

### Fluoroscopy

The DAP was almost the same for Spot ROI and Spot F for FOV = 11 × 11 cm and lower than the DAP for CC. This value for Spot ROI and Spot F was approximately 85% of the value for CC. Using FOV = 15 × 15 cm, the DAP for Spot F was lower than for Spot ROI and CC. For FOV = 15 × 15 cm, the DAP for Spot F was 46% of the DAP for Spot ROI and 75% of the value for CC.

For FOV = 20 × 20 cm, the DAP of Spot ROI was the lowest: 25% of value for Spot F and 16% of value for CC.

The AK for Spot ROI was lower than the AK for Spot F and CC regardless of the FOV. The AK for Spot ROI for FOV = 11 × 11 cm was 44.1% of the AK for Spot F and 37.5% of the AK for CC.

The AK of Spot ROI for FOV = 15 × 15 cm was 33.3% of the AK for Spot F and 22.3% of the AK for CC.

Finally, the AK for Spot ROI for FOV = 20 × 20 cm was 25.1% of the AK for Spot F and 16.9% of the AK for CC. The PSD for Spot F and Spot ROI was similar and lower than the PSD for CC for all FOVs.

The values of PSD for FOV = 11 × 11 cm were 56 mGy for Spot F and 59 mGy for Spot ROI, which were 80 and 84.3%, respectively, lower than the PSD for CC (70 mGy). The values of PSD for FOV = 15 × 15 cm were 47 mGy for Spot F and 46 mGy for Spot ROI, which were 74.6 and 73%, respectively, lower than the PSD for CC (63 mGy). The values of PSD for FOV = 20 × 20 cm were 35 mGy for Spot F and 37 mGy for Spot ROI, which were 64.8 and 68.5%, respectively, lower than the PSD for CC (54 mGy).

### DSA

All measured values of Spot ROI were lower than the corresponding values of Spot F and CC for all three FOVs. The values of these parameters are the same for Spot F and CC since the runs were performed without collimation because the entire FOV was necessary for the creation of the road map. For FOV = 11 × 11 cm, the DAP for Spot ROI was 26.4% of the DAP for Spot F and CC; for FOV = 15 × 15 cm, it was 24.9% of the DAP for Spot F and CC; and for FOV = 20 × 20 cm, the DAP for Spot ROI was 16.5% of the DAP for Spot F and CC.

Similarly, for FOV = 11 × 11 cm, the AK for Spot ROI was 26.4% of the AK for Spot F and CC; for FOV = 15 × 15 cm, it was 24.9% of the AK for Spot F and CC; and for FOV = 20 × 20 cm, it was 20.7% of the AK for Spot F and CC. Finally, for FOV = 11 × 11 cm, the PSD of Spot ROI was 51.5% of the PSD for Spot F and CC; for FOV = 15 × 15 cm, 66.7% of the PSD for Spot F and CC; and for FOV = 20 × 20 cm, it was 75.6% of the PSD for Spot F and CC.

### Fluoroscopy plus DSA

Due to the essential impact of the dose generated by runs (angiographies) on the total dose, parameters of total dose (AK, DAP, and PSD) for Spot ROI were lower than the corresponding parameters for Spot F and CC regardless of the size of the FOV.

## DISCUSSION

While Spot ROI and Spot F show about the same PSD values, AK and DAP differ markedly between the three different modalities. The PSD refers to the patient surface area of the highest cumulative skin dose, which in this case is the area of the central beam. As Spot F and Spot ROI show practically the same X-ray parameters, the same PSD is the logical consequence.

Spot ROI shows the highest DAP value in fluoroscopy mode for FOV 20 and FOV 15 because the exposed field size is significantly larger for SR (Table 1) even though the X-ray intensity is different between the Spot ROI and the surrounding area.

For FOV 11 Spot ROI shows quasi the same DAP than Spot F which could be explained by the lower X-ray parameter used for SR as a result of the ABC response, the smaller difference in effective field size between Spot ROI and Spot F as well as the accuracy tolerance of the DAP meter response to the radiation.

The built-in DAP meter (PTW Diamentor) outputs only the DAP value.

The AK value is calculated by dividing the DAP by the area exposed at the IRP.

In fluoroscopy the area exposed with Spot ROI is ~2.5–8× times larger than the area exposed in Spot F and CC, whereas the DAP difference between Spot ROI and Spot F, CC is only about a factor of 1.5 at max. Hence the resulting AK which takes the area exposed into account is markedly lower for Spot ROI in comparison with Spot F and CC. The difference between Spot F and CC with the identical area exposed can be explained by the different ABC methods used. Spot F uses a similar adaptive ABC technique than Spot ROI which is reflected in the respective X-ray parameters chosen by the system.

These results prove also the importance of a true patient model-based PSD computation and the limitation of AK-based dose values.

It shows that AK-based values can be misleading regarding the actual PSD hitting the patient as AK represents only an average dose value of a homogeneous air field exposed at the IRP, which is 15 cm below system ISO center toward the focus. While the IRP is a fixed point in space, the patient skin entrance plane in contrast is not. It varies with table height, patient thickness, angulation, and isocentric or non-isocentric positioning. Hence the patient skin entrance plane can consequently have either a larger or a smaller distance to the focus than the IRP or even exactly the same distance which results in an over- or underestimation of the PSD when AK is used as reference. Moreover, AK does not take into account PSD relevant and influencing factors such as patient tissue absorption and backscatter characteristics, absorption/scatter effects of table and mattress, the dose distribution pattern, as well as the differences in systems geometry. In conclusion the AK value displayed on the monitor does consequently not give a reliable and correct indication of the actual PSD delivered to the patient. That is why the PSD estimation model used in the Alphenix system is not based on AK as input value but uses a dedicated FDA-approved algorithm for dose computation which is completely separated from the traditional AK calculation using the DAP meter value as input.

The precondition for DSA was to get the entire FOV information. Consequently, in Spot F and CC, no collimation was applied i.e. the effective field size exposed was identical between Spot ROI, Spot F, and CC for all FOVs. CC uses conventional ABC principles like those used in fluoroscopy. The values for Spot F and CC are the same because both use the same ABC technique, resulting in the same X-ray parameters and dose. In contrast, Spot ROI can be applied in all acquisition modalities (fluoroscopy, DSA, digital angiography) i.e. the novel ABC technique described above is also active in DSA. This, in combination with an additional 0.7 mm Cu layer, results in considerably lower dose values for each comparator concerned (AK, DAP, PSD).

The total dose value as the sum of fluoroscopy and DSA is consistent with Spot ROI being the method with the lowest values for AK, DAP, and PSD. Our results showed that Spot F and Spot ROI are superior to CC for each measured parameter regardless of the size of the FOV.

The main advantage of the Spot F in comparison to Spot ROI is that a rectangular or square ROI of any size can be freely moved within the FOV at any time and as often as the operator wants. The FOV, outside the ROI, is completely shielded by collimators. That means, however, that the FOV outside the ROI is hidden and the image displayed on the screen during the fluoroscopy is only the LIH, not a real-time image. The square ROI of constant size generated by Spot ROI can also be moved within the selected FOV. In contrast to the ROI generated by Spot F, the FOV outside this square ROI is still visible, making it possible to track in real time the most important anatomic or device-related information (Figure 2a–c).

### Methodological limitations

There are three limitations of our study. First, statistical analysis of results was not performed since the data we have generated are not appropriate for a statistical evaluation.

We have only one pair of data for each FOV and both acquisition modes (fluoroscopy and DSA). Though the percentage differences between the measured parameters (AK, DAP, and PSD) are obvious, the calculation of *p*-value of these differences would not give meaningful results. Second, the results of our measurements were not compared to results of other studies because studies performed under the same experimental conditions and the same or a similar system have either not been carried out or are not available in the scientific literature. Third, an important limitation of this study is its experimental character. A new study, in a clinical setting, with live cases, is necessary to obtain reliable and comprehensive assessment of the practical benefits of this system. This will be possible since the Spot ROI is integrated into the commercially available ‘Alphenix’ biplane angiographic system.

Finally, evaluation of the Spot ROI under clinical conditions with large number of patients, as already described in the literature with another dose-saving functionality^(9)^, would enable statistical analysis of results which is missing in this study.

## CONCLUSION

Spot ROI is a promising dose-saving technology as it can be applied in fluoroscopy and DSA and digital angiography acquisition. The biggest benefit of Spot ROI is its ability to keep the entire FOV information always visible. Despite limitations, a combination of the use of Spot F and Spot ROI, depending on the clinical situation, is a potentially useful technique for obtaining an appropriate quality of visual information while reducing the radiation dose received by the patient.

## CONFLICT OF INTEREST STATEMENT

The authors declared the following potential conflicts of interest with respect to the research, authorship, and/or publication of this article: The unit for neurointervention in our department is Canon’s reference site. AP is a Canon employee, an engineer, and International Clinical Development Manager.
